# Measuring surface phonons using molecular spin-echo

**DOI:** 10.1039/d2cp01372j

**Published:** 2022-05-26

**Authors:** Helen Chadwick, Gil Alexandrowicz

**Affiliations:** Department of Chemistry, Faculty of Science and Engineering, Swansea University Swansea SA2 8PP UK h.j.chadwick@swansea.ac.uk g.n.alexandrowicz@swansea.ac.uk

## Abstract

A new method to measure surface phonons with a molecular beam is presented. The method extends the principles of ^3^He spin-echo spectroscopy, to the more complex case of a molecular beam exchanging energy with the surface. Measurements are presented for inelastic scattering of D_2_ from a Cu(111) surface. Similarly to helium spin-echo, experiments can be performed along optimal tilted projections making it possible to resolve energy peaks with a high energy resolution which is not restricted by the spread of energies of the incident beam. Two analysis methods for these molecular spin echo experiments are presented. A classical approach, analogous to that used for helium spin-echo, explains the most dominant excitation peaks measured, whereas a semi-classical approach allows us to identify smaller peaks which are related to the complexity of the multiple spin-rotation states which exist for molecules.

## Abstract 2

A new method to measure surface phonons with a molecular beam is presented.

## Graphical abstract

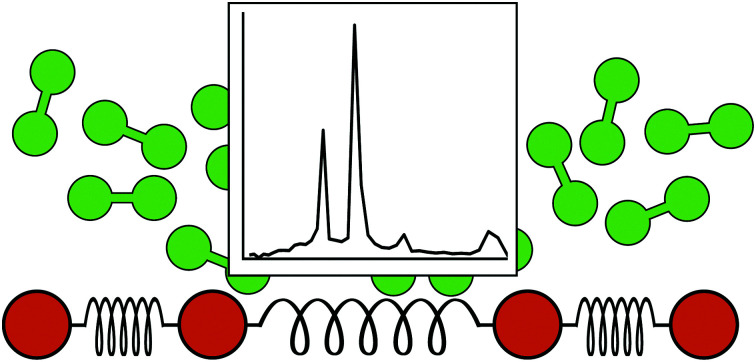

## Introduction

Inelastic scattering measurements using atomic and molecular beams provide unique insight into the atomic scale dynamics of surfaces.^1^ Furthermore, essentially any surface chemistry process is affected, and in many cases dominated, by the ability of molecules to exchange energy with the surface.^2^ Surface phonons often provide an important channel for this energy exchange, making them particularly important to investigate.

Helium atoms are an excellent choice for studying surface phonons, and the majority of helium atom scattering (HAS) experiments which characterised phonons and their dispersion curves were performed using the time-of-flight (TOF) technique. While a great deal of our understanding of low energy phonon modes is the result of HAS TOF experiments, this technique has its limitations. One of the fundamental restrictions on the achievable resolution in TOF experiments is the velocity spread of the incident beam, since the measured spectra are a convolution of the real excitation spectra with the energy distribution of the beam. This restriction has been addressed by improving supersonic expansions, where helium beams with very narrow energy distributions have been successfully produced and relatively high resolution spectra were obtained from numerous surfaces.^1^ Nevertheless, in various circumstances, for example when trying to separate two phonon modes which are close in energy, when trying to separate a very low energy mode from the elastic peak, or when we are attempting to measure the true linewidth of a phonon, the fact that the resolution of TOF experiments is on the order of a fraction of a meV can become a significant problem. Other contributions broadening the spectrum further are related to the chopper width, detector time response and other experimental restrictions, all of which have been minimised over the years by optimising TOF instruments.^3,4^

A way to circumvent the incident energy broadening effect is offered by the helium spin-echo (HSE) technique. HSE uses ^3^He beams which have a nuclear spin *I* = ½, and similarly to neutron spin-echo, the precession of the spin in magnetic fields before and after scattering offers an alternative way for measuring energy changes due to the scattering event. Unlike TOF measurements, within a first order approximation, the velocity spread of the incident beam does not broaden the spectrum, which leads to a dramatic improvement in energy resolution, especially when using beams with a relatively wide energy spread, allowing reconstruction of phonon spectra in the μeV range.^5^ The main application of the HSE technique is for quasi-elastic scattering studies used to detect and characterise surface diffusion,^6^ however, it can also be used to study inelastic scattering in general, and surface phonons in particular. Using a simple classical model, termed the tilted projection picture^7^ for designing and interpreting the measurements, ultra-high resolution measurements of surface phonons have been used to measure the low energy part of dispersion curves, anharmonicity, and phonon lifetimes of a metal surface.^5,7^ Helium spin-echo measurements of surface vibrations have also been used to determine the dispersion relations and lifetimes of adsorbate vibrations,^8–10^ and even phasons.^11^

While often involving a very similar setup to HAS, molecular scattering experiments offer quite different and complimentary insight to atom scattering. The inertness of helium atoms makes them an ideal probe for studying various surface structure and dynamics problems in a way which is to a good approximation independent of the properties of the probe.^12^ However, when it comes to studying the interaction of molecules with surfaces, which is one of the main goals of surface science, molecular scattering experiments provide insight into the full interaction potential and the surface chemistry taking place.^13,14^

Using molecular beams to study surface phonons is more of a rarity. Early works have demonstrated that not only is this possible, but molecular beams also offer unique insights due to their ability to detect surface phonons which are invisible to HAS due to their polarisation and symmetry constraints.^15^ Another potential advantage of studying surface phonons with molecular beams, is that it provides the most direct way of studying energy loss processes for a molecule colliding with a surface. Losing energy is an essential step in the trapping of a molecule on a surface, and for low energy molecules it directly impacts the probability of a reaction on a surface.^16–20^ Performing high resolution studies of surface phonon excitations by molecular beams, would provide experimental benchmarks for developing theoretical modelling of chemisorption beyond the Born Oppenheimer static surface approximation.

In this manuscript we demonstrate that the basic principles which enable phonon measurements with HSE and are used to interpret the results, can be extended to a molecular beam, and be used to reconstruct spectra with energy resolutions comparable to those achievable with HSE. We start by explaining the principle of this new type of measurement within the simple classical picture of tilted projections, show experimental results obtained from scattering a D_2_ molecular beam from a Cu(111) surface, and then present a more thorough method for simulating the spectra and interpreting the measured peaks.

## Experimental

**Fig. 1:**
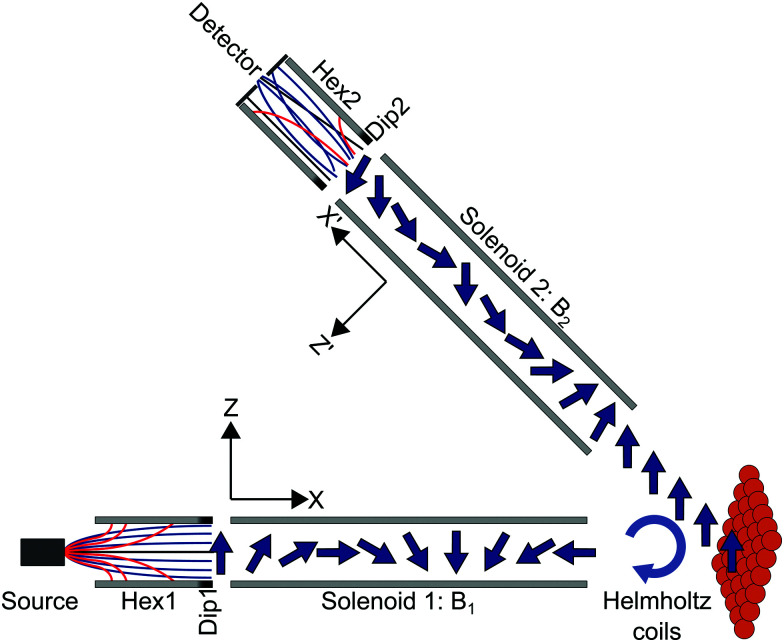
Schematic of the experimental apparatus showing the positions of the various magnetic elements that are used to manipulate the magnetic moments of the D_2_ molecules (represented as arrows) and the frames of reference for propagation down the first and second arm of the machine. Hex1, Dip1/Hex2, Dip2 mark the positions of the first/second hexapole magnets and the dipolar fields positioned after/before the hexapoles.

In the experiments presented here (apparatus shown schematically in Fig. 1), the molecular beam of D_2_ first travels through a magnetic hexapole^21^ which creates an inhomogeneous magnetic field which either focusses or defocusses the D_2_ molecules depending on the rotational (*m*_*J*_) and nuclear spin (*m*_*I*_) projection state of the molecule. At the end of the hexapole, there is a hexapole to dipole element which adiabatically rotates the projection states from the various field directions within the hexapole to that of the dipole, which defines the quantisation (*z*-) axis in the first arm of the machine. After a field free region, the molecules enter the first solenoid which creates a tuneable magnetic field (*B*_1_) perpendicular to the initial quantisation axis and is either parallel or anti-parallel to the beam propagation direction for negative and positive field values respectively. In the classical picture, this causes the magnetic moments to precess with a Larmor frequency which is determined by the value of the gyro-magnetic ratio and the magnetic field, which is controlled by changing the current that is passed through the solenoid. Quantum mechanically, the magnetic field leads to Rabi oscillations of the populations of the molecules between the different *m*_*I*_, *m*_*J*_ states. The molecules then pass through a Helmholtz coil, located at the entrance to the scattering chamber, which can be used to generate a small field along the *y*-axis (see Fig. 1) just before they collide with the Cu(111) surface.††The Helmholtz coil produces a field which is perpendicular to the *B*_1_ direction and is used to compensate for the scattering geometry and change the relative intensity of parallel and anti-parallel echoes. It should not be confused with the phase coils used in NSE, used to scan the spin-echo condition. After scattering, a fraction of the molecules enter the second arm of the machine where there is a second solenoid which creates a tuneable field *B*_2_, followed by a second hexapole,^22^ both of which manipulate the molecules in the same way as in the first arm. At the end of the second arm there is a custom-built particle detector which ionises the molecules and measures the corresponding current that is produced.^23^

There are various ways to design and interpret a HSE measurement, one relatively simple way of understanding how we can measure energy changes which are much smaller than the energy distribution width of the incident beam, is the tilted projection picture.^7^ Below we give a brief description of this picture, more detailed explanations can be found in previous publications.^5–7,24^

If the nuclear spins are treated as classical magnetic moments which undergo Larmor precession in the two electromagnets (see Fig. 1), it can be shown that a two dimensional measurement of the scattered intensity as a function of the strength of the two magnetic fields, *S*(*B*_1_,*B*_2_), is proportional to the Fourier transform of the 2d wavelength matrix, *ρ*(*λ*_i_,*λ*_f_).^5,7,24^ The wavelength matrix expresses the probability of having a beam particle with an initial de-Broglie wavelength, *λ*_i_, which scatters from the surface with a specific final wavelength, *λ*_f_. The fact that *ρ*(*λ*_i_,*λ*_f_) is a 2d function, means that a wide distribution of initial energies (wavelengths) doesn’t lead to any loss of information, as each element of the matrix ‘stores’ the information for an initial and final energy (wavelength) independently. While this matrix contains all the information about the scattering process, to the best of our knowledge, it has only been reconstructed directly from experimental data once,^24^ due to the extremely time-consuming process of performing a 2d measurement with sufficient resolution and range.

Even if reconstructing *ρ*(*λ*_i_,*λ*_f_) is usually impractical, it is a useful concept because of the Fourier projection theory, which tells us that if we perform a 1d experiment where *S*(*B*_1_,*B*_2_) is measured by changing the magnitude of the two fields while maintaining a proportionality condition *B*_1_ = *αB*_2_, and Fourier transform the signal, the result is a projection of *ρ*(*λ*_i_,*λ*_f_) onto an axis which has the same angle in wavelength space. While experiments can be performed at any angle by changing *α*, some angles are more useful than others as we will explain below and later demonstrate experimentally.

Elastic, quasi-elastic or inelastic scattering events define specific relations between the incoming and scattered wavelengths. Within a first order approximation this can be thought of as a straight-line segment in the *λ*_i_,*λ*_f_ plane. The length of the linear segment is determined by the width of the distribution of incident wavelengths. The width of the linear segment is given by the actual linewidth of the excitation (for example this could be quasi-elastic broadening due to diffusion, or phonon width broadening due to the finite lifetime of the phonon). Finally, the position and angle of the linear segment within the *λ*_i_,*λ*_f_ plane depend on the specific excitation. As a result, if we want the distribution width of the incident energies to not broaden the spectrum of a 1d measurement, the projection angle should be set such that it is perpendicular to the slope of the line. Within the approximation that the excitation feature in the *λ*_i_,*λ*_f_ plane is linear, an approximation which becomes better for beams with a relatively narrow energy spread, this allows reconstruction of the excitation peak without any broadening due to the different incident energies.

In order to extend the tilted projection picture from ^3^He beams to molecular beams, a few complexities need to be considered. One source for complications are multiple echoes. Even for the simple case of ^3^He, a spin ½ particle, the tilted projection picture, as well as all other previous derivations of neutron and helium spin-echo techniques the authors are aware of, do not account for the simultaneous existence of parallel and anti-parallel echoes.^25^ The existence of these two types of echoes leads to mirror like features in *S*(*B*_1_,*B*_2_) which depend on the total scattering angle of the apparatus used. Fortunately, this complication can be mostly resolved by using an additional rotation coil before scattering which compensates for the scattering geometry and can be used to isolate one of the two echo conditions. In our experimental system this would be the Helmholtz coil located at the entrance to the scattering chamber (Fig. 1).

The phenomena of multiple echoes becomes much more involved for molecular systems as they are multi-level quantum systems, with multiple conditions for constructive interference and correspondingly a large number of spin-echo features.^26^ To accurately simulate the signal of a molecular scattering experiment, even for the simpler case of elastic scattering, a quantum/semi-classical calculation is needed as well as a scattering matrix, describing the changes of the wave-functions due to scattering.^26^ Nevertheless, as we will show below, if we are only interested in identifying peaks within the energy spectra, the classical tilted projection can still be used, as long as the gyro-magnetic ratio is adjusted to the energy splitting of the multi-level system, and the existence of multiple eigen-energies and multiple spin isomers is taken into account.

The particular molecule used in the experiments presented below is D_2_. The interaction of dihydrogen, which is the most abundant molecule in the universe, with surfaces is important for a wide range of research fields and chemical applications and the various isotopes of dihydrogen are commonly used in many surface science studies.^27–29^ D_2_, is also a good choice for performing the first molecular spin-echo measurements of phonons, as it has a non-rotating state with 5 spin projection states, *J* = 0, *I* = 2, which produces relatively simple spectra.

**Fig. 2:**
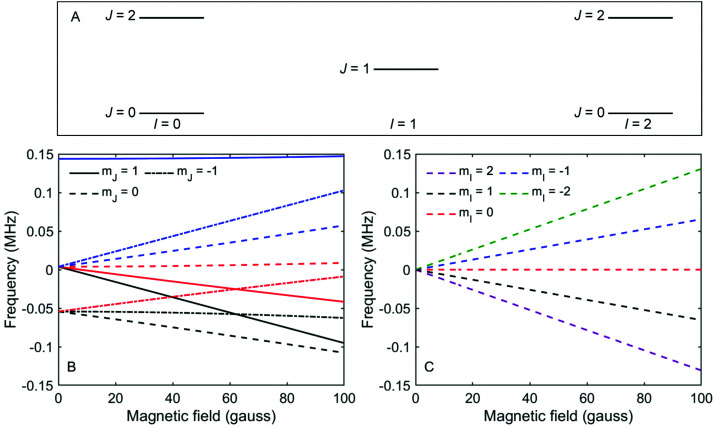
(A) Schematic energy level diagram showing the three lowest rotational states (*J*) and corresponding nuclear spin states (*I*) in a D_2_ molecule. (B) The energy of the different *m*_*I*_, *m*_*J*_ states of D_2_ molecules in *I* = 1, *J* = 1, as a function of magnetic field.^30^ The colours of the lines correspond to the *m*_*I*_ state as defined in the legend of panel (C). (C) The energy of the different *m*_*I*_, *m*_*J*_ states of D_2_ molecules in *I* = 2, *J* = 0, as a function of magnetic field.

Fig. 2A shows a schematic energy diagram of the 3 lowest rotational, *J*, states in D_2_, and which total nuclear spin states are allowed for these *J* states. Fig. 2B and C show the energy splitting in a magnetic field for the *J* = 1, *I* = 1 and *J* = 0, *I* = 2 states respectively.^30^ The other allowed *J* = 2 state (*J* = 2, *I* = 2 and *J* = 2, *I* = 0) splits into 30 levels and interested readers are directed to Fig. 3A in ref. 31 for the energy dependence of these states as a function of magnetic field. What is evident from Fig. 2B and C is that the rotational projection states, and the corresponding rotational spin coupling which exist in *I* = 1, *J* = 1 lead to non-linear energy dependencies on magnetic field with multiple transition frequencies.^30^ In contrast, in *J* = 0, *I* = 2, there are only 5 spin projection states, the energy dependence on the magnetic field is linear, and the transition frequencies for a given B value are simply integer multiples of the same fundamental frequency. It should be noted that the *J* = 0, *I* = 0 state cannot be manipulated by magnetic fields and therefore contributes a constant background to the measurements that are presented here.

**Fig. 3:**
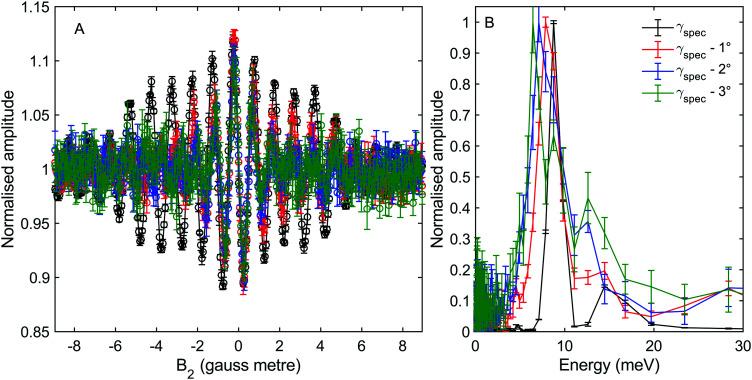
(A) Oscillation curves measured at different values of incident angle corresponding to specular scattering (*γ*_spec_, black), *γ*_spec_ − 1° (red), *γ*_spec_ − 2° (blue) and *γ*_spec_ − 3° (green) as a function of the integral magnetic field in the second arm of the machine, *B*_2_. *B*_1_ was fixed at 0 gauss metre. (B) The energy spectra reconstructed from the measurements presented in panel (A).

The experimental apparatus used in the current study has been described in previous work,^26,31–33^ and only the details relevant for the this work are presented here. The molecular beam of D_2_ was formed from a supersonic expansion created using a 20 μm diameter nozzle held at 40 K and passing it through a 0.5 mm diameter skimmer. After travelling through the magnetic hexapole and solenoid in the first arm of the machine as described above and shown schematically in Fig. 1, the beam hits a Cu(111) surface (surface preparation laboratory) held at a temperature of 300 K, oriented such that the crystal 〈11−2〉 azimuth is within the scattering plane with an accuracy of ±0.05° determined from the position of the diffraction peaks in a D_2_ scattering experiment. After scattering, the molecules pass through the second solenoid and hexapole as described above, before the signal is measured using a custom built detector, designed by the surface physics group in Cambridge.^23^ The current through both solenoids is controlled using high stability power supplies (Danfysik) which allows each of the two to be scanned independently or whilst maintaining a fixed ratio between the two fields which they produce.

Fig. 3A shows measurements of the scattered intensity while maintaining *B*_1_ = 0 gauss metre‡‡We use magnetic field integral rather than magnetic field, as the former governs the accumulated effect of the magnetic field on the phase of the magnetic moment. and scanning only *B*_2_. This type of measurement is denoted a 90° tilted projection angle, it is relatively easy to perform (no need to maintain an accurate current ratio between the two coils), and is in many ways equivalent to a TOF measurement, *i.e.*, it has an energy resolution which is limited by the energy distribution width of the incident beam. The signal oscillates in all four measurements reflecting the changes to the quantum states of the molecules which pass through the magnetic field *B*_2_ which lead to different transmission probabilities through the hexapole analyser into the particle detector. We can also see that the frequency of the oscillation changes as a function of the incident scattering angle, indicating different scattered energies. The simplest way to analyse the signal is to follow the procedure developed for ^3^He,^5–7,24^ replacing the gyro-magnetic ratio of ^3^He with the relevant gyro-magnetic ratio for deuterium. Unfortunately, there is not a single gyro-magnetic ratio which characterises D_2_, as there are multiple levels. Furthermore the magnetic dependence of the states is non-linear for *I* = 1, *J* = 1 and for both *I* = 0 and *I* = 2, *J* = 2, rendering the concept of a gyromagnetic ratio, less useful. However, as we will show below, the majority of the signal in our experiments will be from the *I* = 2, *J* = 0 state and the frequency which predominantly modulates the signal is equivalent to the fundamental transition energy between adjacent eigen-energies (the separation between the 5 different eigen-energies plotted in Fig. 2C). These simplifying circumstances, make it possible to analyse the signal quite accurately using a single gyro-magnetic ratio of 653.6 Hz gauss^−1^.^30^ A more accurate approach which considers the various frequencies is presented later in this manuscript.

**Fig. 4:**
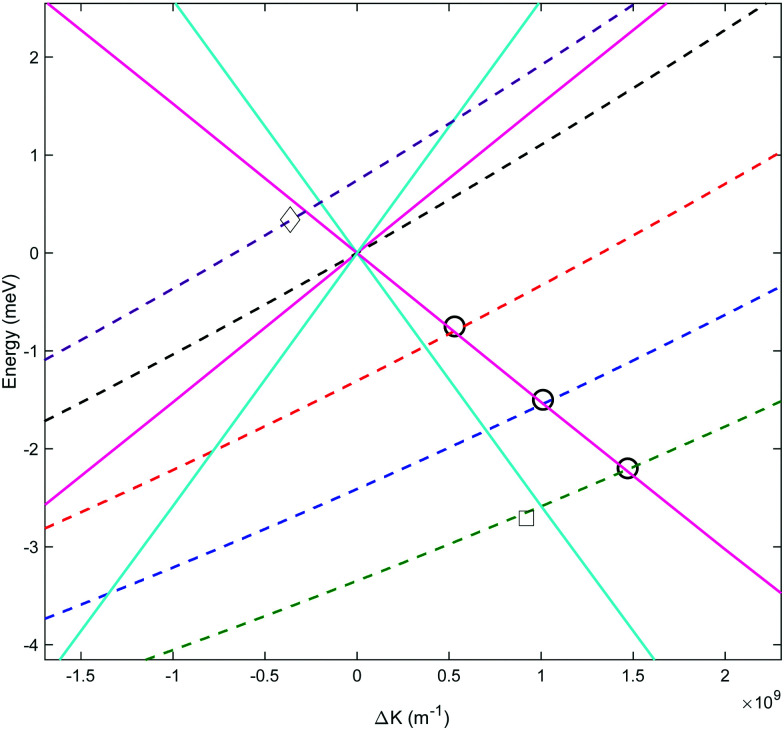
Phonon dispersion curves showing the momentum transfer and energy changes from sinusoidal fits to previous measurements^34^ of the Rayleigh (solid magenta) and longitudinal (solid cyan) phonons, and their intersection with scan curves for *γ*_spec_ (dashed black), *γ*_spec_ − 1° (dashed red), *γ*_spec_ − 2° (dashed blue), *γ*_spec_ − 3° (dashed green) and *γ*_spec_ + 0.5° (dashed purple) incident conditions. The circles are the Δ*E*(Δ*K*) values corresponding to the dominant energy loss peaks we measured at *γ*_spec_ −1°, −2° and −3°. The square marker corresponds to the weaker energy loss peak attributed to the longitudinal mode which became resolvable when using an optimal tilted projection angle. The diamond marker corresponds to the energy gain peak measured at *γ*_spec_ + 0.5°, also using an optimal tilted projection angle.

Fig. 3B shows the spectra reconstructed from these four measurements when we assume classical precession with a gyro-magnetic ratio of 653.6 Hz gauss^−1^.^30^ The black line, which was measured on the specular scattering peak (*γ*_spec_), is dominated by a peak at 8.6 meV. For specular conditions we expect elastic scattering to dominate, and the peak is a measurement of the energy distribution of the incident beam. The FWHM of the peak is approximately 2 meV, reflecting a rather wide incident energy distribution for the nozzle expansion conditions used in the experiment. The largest peaks in the spectra of the −1°, −2° and −3° off specular measurements appear at energy losses, Δ*E*, of 0.75, 1.5 and 2.3 meV with respect to the elastic peak, corresponding to a change of the wave-vector within the surface plane, Δ*K*, of 0.06 Å^−1^, 0.11 Å^−1^ and 0.14 Å^−1^ respectively. These Δ*E*(Δ*K*) values are in good agreement with the dispersion relation of the Rayleigh mode determined from TOF HAS by Harten *et al.*^34^Fig. 4 shows these 3 energy loss peaks as circle markers, alongside a sine fit to the experimental results Harten *et al.*^34^ measured for the Rayleigh mode (solid magenta line). The black, red, blue and green dashed lines show the scan curves (conditions for conservation of energy and momentum) for *γ*_spec_ and the off-specular angles of −1°, −2° and −3°. These scan lines also have other intersections with the Rayleigh mode; however, they have significantly larger energy losses and gains and are less likely to be excited with a high enough probability to appear in the experimental spectra.

Harten *et al.*,^34^ showed that helium atoms can also excite or de-excite the longitudinal mode of this surface. A sine fit to the TOF results for this mode is plotted in Fig. 4 using a cyan line. The scan curves of the relatively small off-specular-angle measurements we performed, cross the dispersion curve of this phonon at energy losses which are only shifted by 150 μeV to 400 μeV with respect to the Rayleigh mode. Given the relatively large width of the Rayleigh mode peak in the measurement, which is predominantly due to the incident energy spread, we do not expect to resolve this excitation. Later, we show a type of measurement which can resolve the two modes even at these low energies.

We also note that there is another peak which appears above the noise in the spectra of all the measurements, in the region between 12–16 meV. The peaks within this region illustrate the complications associated with analysing molecular spin-echo measurements and could have been interpreted erroneously as due to energy gain scattering. These peaks are actually related to scattering of *I* = 1, *J* = 1 molecules, as we will later show using a semi-classical simulation of the experiment.

**Fig. 5:**
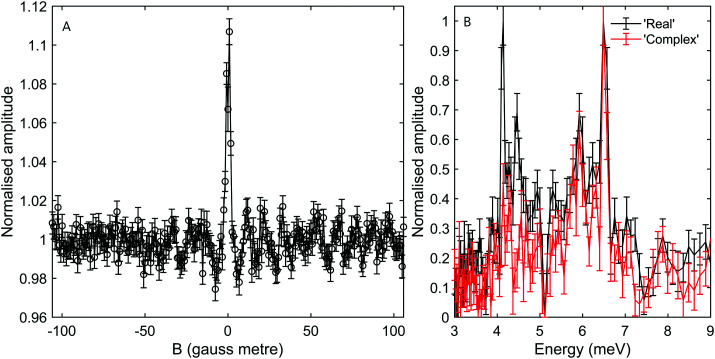
(A) Oscillation curve measured while maintaining the condition *B*_1_ = 1.3*B*_2_, where *B* corresponds to the magnitude of the total magnetic field integral 
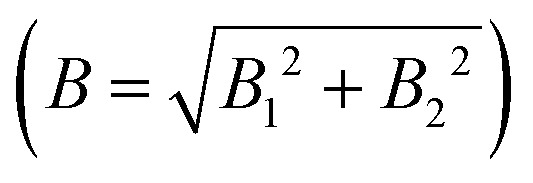
, at an incident angle of *γ*_spec_ − 3°. (B) The energy spectrum derived from the measurement presented in panel (A) (black) and from a measurement of the ‘complex’ signal (red). The energy of the incident beam is centred at 8.6 meV, and the two peaks which dominate the spectra reconstructed from the complex signal correspond to energy losses of 2.1 and 2.7 meV.

While the *B*_2_ scans and calculated spectra shown in Fig. 3 already supply us with information about the dispersion curve, the finer details of the spectra cannot be resolved due to the convolution with the wide energy distribution of the incident beam. In particular it would be useful to be able to separate the longitudinal and Rayleigh modes, which is difficult if not impossible with this particular type of tilted projection measurement. However, if we perform measurements using a tilt angle which is optimised to eliminate the broadening due to the incident energy spread, this becomes possible. Fig. 5B shows a measurement performed −3° off specular using a tilt angle of 52.4°, *i.e.*, scanning the field magnitude while maintaining *B*_1_ = 1.3*B*_2_. Fig. 5B shows the corresponding reconstructed spectrum. The two main peaks in the spectrum which can be seen at 6.5 meV and 5.9 meV are related to the dominant Rayleigh mode and a second weaker, but still clearly resolvable, energy loss attributed to the longitudinal phonon. The ability to clearly differentiate two energy loss peaks which are separated by a fraction of the incident beam energy distribution width, nicely demonstrates the power of the tilted projection approach. The position of the second peak which became apparent due to the optimal tilted projection is plotted using a square marker in Fig. 4 and agrees well with the sine fit to the TOF measurements of the longitudinal mode. Note that the longitudinal mode energy losses measured previously using HAS TOF^34^ were restricted to the range |Δ*E*| > 4 meV, correspondingly, the small mismatch between the peak we measured and the sine fit to the previous data, is not surprising.

It is also important to note that there are complications when using optimal tilted projections, instead of a simple *B*_2_ scan. When measuring the magnetisation (spin projection) only along one axis, defined by the direction of the dipolar field before the second hexapole, the Fourier transform relation between *ρ*(*λ*_i_,*λ*_f_) and *S*(*B*_1_,*B*_2_) becomes a cosine transform, giving rise to spurious reflection peaks on the counter side of wavelength axis.^5,7,24^ For the particular tilted projection angle we chose, the wavelength axis origin is located at 5.12 meV and correspondingly the spectrum shown in black in Fig. 5B, which is derived only from the measurement presented in Fig. 5A, contains what looks like lower energy peaks, which are simply reflections of the two modes discussed above.

To remove the mirror image peaks, it is necessary to measure the magnetisation along a perpendicular axis (*y*′-axis) to the original (*z*′-axis), which would produce a signal with a 90° phase shift compared to the first measurement.^5,7^ This can be achieved to a reasonable approximation by performing two measurements where the value of *B*_1_ is alternated throughout the scan for each value of *B*_2_ to introduce a phase shift of 90° between the two sets of data, one of which can then be considered as the ‘real’ component *S*_r_(*B*_1_,*B*_2_) and the other the ‘imaginary’ component *S*_i_(*B*_1_,*B*_2_). A ‘complex’ signal can then be defined as 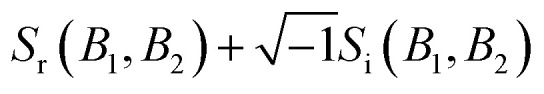.^5,7^ This procedure was performed for the tilted projection measurement presented in Fig. 5, and the energy spectrum that was derived is presented as a red line in Fig. 5B. As can be seen, this spectrum no longer has significant mirror symmetry around 5.12 meV and allows us to differentiate the true peaks from the mirror images in the original spectrum.

**Fig. 6:**
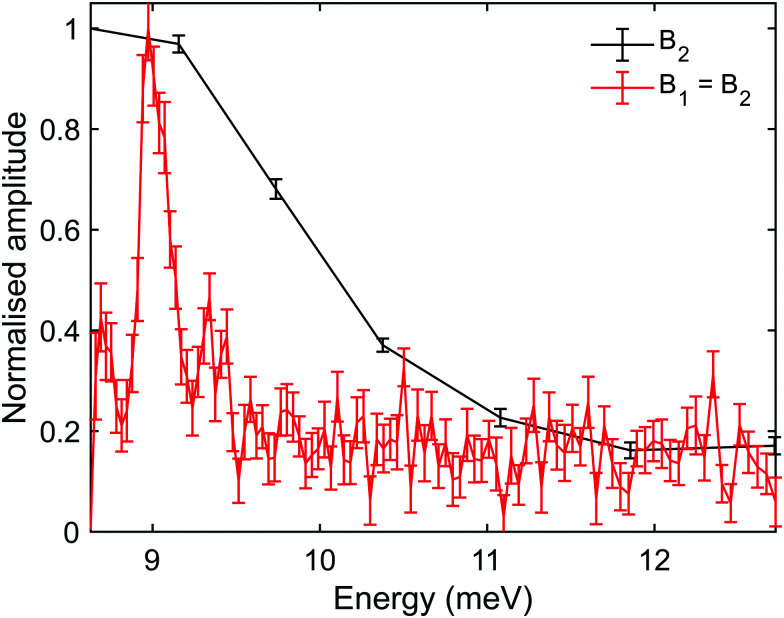
Comparison of the energy spectra obtained at an incident angle of *γ*_spec_ + 0.5° when scanning *B*_2_ and holding *B*_1_ at 0 gauss metre (black line), and when scanning along the tilted projection *B*_1_ = *B*_2_ condition (red line). The vertical scale has been truncated to focus on the phonon peak, the elastic peak at 8.6 meV is a delta function (for a 45° tilted projection experiment) and is approx. 500 times more intense than the phonon peak.

Another scenario when optimised tilted projections are particularly useful, is when the energy gain/loss is very small, and it is expected to merge within the diffuse elastic scattering peak, unless we can perform a measurement which decouples the two. Fig. 6 shows measurements performed only +0.5° from specular using tilt angles of 45° (*B*_1_ = *B*_2_) and 90° (*B*_1_ = 0). The energy gain peak in this case is only shifted by 350 μeV from the elastic peak at 8.6 meV and yet can be seen clearly in the spectrum of the 45° tilted projection measurement (red line). This energy gain corresponds to a Δ*K* of −0.036 Å^−1^, and is shown using the diamond marker in Fig. 4. The particular optimal tilted projection we used (45°), projects a pure elastic peak into one bin, the origin of the wavelength axis.^7^ The intensity of the diffuse elastic peak is approximately two orders of magnitude larger than the intensity of the phonon excitation peak, and the vertical scale in Fig. 6 is truncated to allow us to see the phonon peak.

In contrast, a tilted projection of 90° (black line), simply shows one broad peak which is a combination of the diffused elastic scattering and the (much weaker) inelastic peaks. The fact that the phonon energy is smaller than the width of the incident beam, combined with the overwhelming intensity from the diffused elastic peak when measuring so close to the specular peak, makes this type of measurement uninformative. Furthermore, our calculation predicts that for a 45° tilted projection, the broadening related to the incident energy beam widths is negligible, hence the full width half maximum of the peak (approx. 200 μeV) is related to the lifetime of the phonon and/or the merging of the Rayleigh and longitudinal mode for these extremely small energy gains (large wavelengths).

## Simulations

In the analysis of the results above, we adapted the classical tilted projection method, originally developed for ^3^He to the case of D_2_. While this was sufficient to explain the main features seen in the spectra, it is also a rather simplistic approach. In particular, as was shown for elastic H_2_ scattering measurements,^32,33^ the signal oscillations includes many different frequencies, which are ignored in the tilted projection analysis we presented above. To provide a better level of analysis we modified a semi-classical simulation method which was developed for elastic H_2_ experiments,^32,33^ to include the change of energy after scattering. These semi-classical simulations solve the spin and rotational degrees of freedom quantum mechanically and treat the translation of the molecule classically. Below we briefly describe the simulation method and then show the simulated spectra it produces.

To simulate the signal, the effect of each magnetic element, shown schematically in Fig. 1, on the D_2_ molecules needs to be calculated. The probability that an initial *m*_*I*_, *m*_*J*_ state (*n*) which has an initial wavelength *λ*_i_ is transmitted through the first hexapole (*P*_hex,1_(*n*,*λ*_i_)) is determined using semi-classical trajectory deflection calculations.^35^ Due to the strong magnetic field gradients, the superposition states decohere, meaning that at this point the states can be described as pure *m*_*I*_, *m*_*J*_ states^36^ where the quantisation axis is taken as the direction of the dipole at the end of the hexapole (the *z*-axis). The internal states are then propagated coherently through the measured magnetic field profile of the first arm of the machine by quantum mechanically solving the magnetic Hamiltonian 
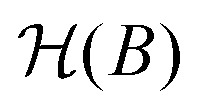
.^30^ The evolution of the quantum states through the first arm of the instrument can be described using a propagation matrix *U*(*B*_1_,*λ*_i_),^32^ where *λ*_i_ has been included explicitly as we are considering inelastic scattering, which when operated on the initial quantum state gives us the molecular quantum state just before the scattering event.

The molecules then collide with the surface which can result in an energy and momentum change of the D_2_ depending on whether it excites or de-excites a phonon, and the scattering geometry. In the calculations below, the wavelength after scattering, *λ*_f_, was chosen to fit the phonon dispersion curves for this surface determined by Harten *et al*.^34^ It is assumed that the *m*_*I*_, *m*_*J*_ state is not changed by the collision with the surface. Assuming *m*_*I*_, doesn’t change is likely to be a good approximation for the *m*_*I*_ states of the molecules as the copper surface is non-magnetic. Whilst the *m*_*J*_ states in the *I* = 1, *J* = 1 state could, and generally speaking would be changed by the collision,^32^ this will change the relative intensities of the different frequency components of the signal but not the frequencies themselves. As it is the frequencies which will be used to identify the features in the spectra, and as we do not have any predictions for the scattering matrix values, we will make the simple choice of Δ*m*_*J*_ = 0, keeping in mind that we do not expect the relative intensities of the peaks in the simulated spectra to be realistic.

The *m*_*I*_, *m*_*J*_ states after scattering are propagated through the measured magnetic field of the second arm of the machine in the same way as the first, *i.e.*, using the Hamiltonian given in ref. 30 to give the propagation matrix *U*(*B*_2_,*λ*_f_)^32^ where *λ*_f_ accounts for the different velocity that the molecules have after the inelastic scattering event, and the transmission probabilities of the final *m*_*I*_, *m*_*J*_ state (f) through the second hexapole (*P*_hex,2_(*f*,*λ*_f_)) are again found using semi-classical trajectory bending calculations. The wave-function of the molecules in an initial *m*_*I*_, *m*_*J*_ state which are in a final state at the detector (|*ψ*_*fn*_〉) can therefore be written as1
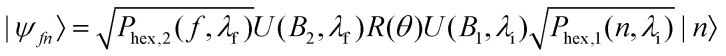
where *R*(*θ*) is a rotation matrix which accounts for the angle between the two arms of the machine and effectively changes the quantisation axis from the *z*-axis for the propagation down the first arm of the machine to the *z*′-axis for the propagation down the second arm of the machine (see Fig. 1). The signal, (*B*_1_,*B*_2_), for a given energy change can then be calculated using2
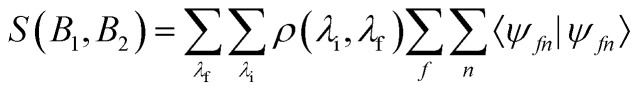
where *ρ*(*λ*_i_,*λ*_f_) accounts for the different weights of the wavelengths in the initial molecular beam and the scattered molecules, and the sums run over the final wavelengths, initial wavelengths, final states and initial states, where the initial states have been weighted to account for the relative abundance of the *I* = 1, *J* = 1 and *I* = 2, *J* = 0 states in the molecular beam expansion, *i.e.*, 3/9 : 5/9 (the *J* = 2 state will not be significantly populated at a nozzle temperature of 40 K). The remaining 1/9 of the beam in the *I* = 0, *J* = 0 state is not included in the calculations as it only contributes to a constant background signal.

**Fig. 7:**
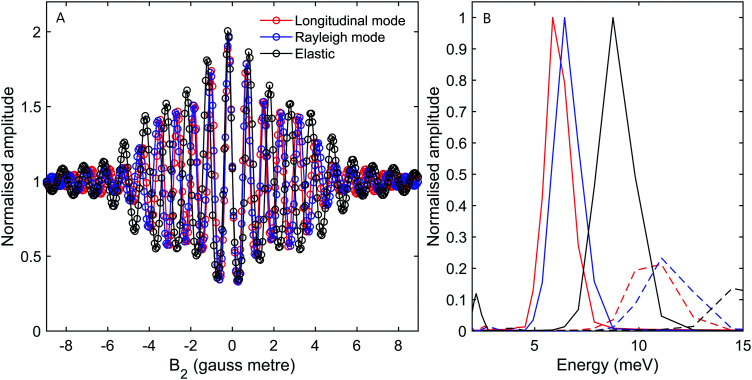
(A) Oscillation curves calculated for a scattering angle of *γ*_spec_ − 3°. The calculations were performed for the energy losses expected from the longitudinal mode (red markers), the Rayleigh mode (blue markers) and for elastic scattering (black markers). The simulations were performed for a 90° tilted projection angle (scanning just *B*_2_). (B) The energy spectra reconstructed from the calculations presented in panel (A), using the same colour scheme. Dashed lines mark spectra calculated for the *I* = 1, *J* = 1 state.

**Fig. 8:**
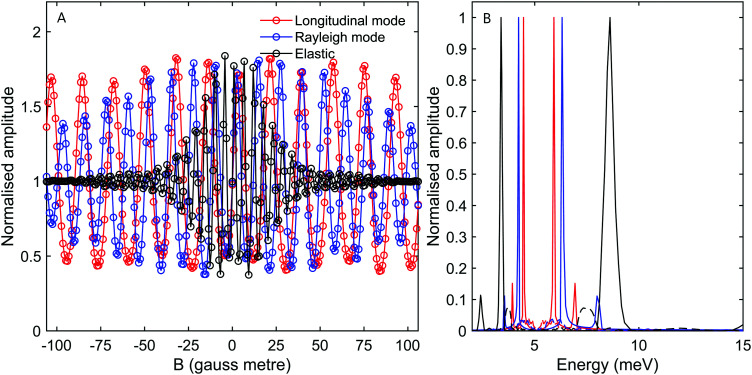
(A) Oscillation curves calculated for a scattering angle of *γ*_spec_ − 3°. The calculations were performed for the energy losses expected from the longitudinal mode (red markers), the Rayleigh mode (blue markers) and for elastic scattering (black markers). The simulations were performed for a magnetic field ratio *B*_1_ = 1.3*B*_2_ corresponding to a tilt angle of 52.4°. (B) The energy spectra reconstructed from the calculations presented in panel (A), using the same colour scheme. Dashed lines mark spectra calculated for the *I* = 1, *J* = 1 state.

Signals given by eqn (2) were calculated for various conditions. In addition to calculating the case of elastic scattering, two different energy losses were simulated. These were calculated from the intersection of the scan curve consistent with a *γ*_spec_ − 3° experiment with the fitted Rayleigh and longitudinal modes (see Fig. 4). Note that due to the significant width of the energy distribution of the incident beam, different scan curves and correspondingly different intersections with dispersion curves, need to be taken into account. Calculations were performed for magnetic field scans which correspond to 90° and 52.4° tilted projection measurements. The traces in Fig. 7A and 8A show simulated signals for the different values of Δ*E* at tilted projection angles of 90° and 52.4° respectively (note the different *x*-axis scales on these two plots).

To understand the impact of analysing molecular spin-echo phonon measurements within the simple classical, single gyro-magnetic tilted-projection picture, we can apply the type of analysis used to interpret the experimental signals on the simulated signals and obtain reconstructed spectra. Fig. 7B, shows the reconstructed spectra for simulations of elastic scattering (black) and energy losses from Rayleigh (blue) and longitudinal (red) mode excitations. The solid lines are calculations of *I* = 2, *J* = 0 molecules and dashed lines are for *I* = 1, *J* = 1 molecules. While the simple reconstruction procedure for the *I* = 2, *J* = 0 molecules produces peaks at the expected positions, for *I* = 1, *J* = 1, the elastic peak and the energy loss peaks all appear as if they are energy gains. The position of these peaks suggests that the origin of the small but still significant peaks within the 12 meV to 16 meV range seen in Fig. 3B is from non-negligible *I* = 1, *J* = 1 contributions. Fig. 8B shows a similar type of analysis, this time for a different tilted projection angle (52.4°). Again, *I* = 1, *J* = 1 produces small but non-negligible contributions which appear this time at around 7 meV. The reconstructed spectrum in Fig. 8B, which is characterised by very narrow peaks for both the Rayleigh and longitudinal modes, clearly demonstrates the advantage of using an optimal tilted projection to obtain a higher resolution spectrum and differentiating closely spaced excitation peaks.

The signals and spectra presented in Fig. 7 and 8 were calculated from eqn (2), using a simple identity scattering matrix to describe the changes in the wave-function during the scattering event, whereas changes to the velocity of the scattered molecules were chosen to fit the dispersion relation of the phonon mode simulated. The results of such calculations, allow us to identify the frequencies which the signal is composed of and associate them with *I* = 2, *J* = 0 and *I* = 1, *J* = 1 states and elastic and inelastic scattering contributions. As a result, we can identify the peaks which appear in the reconstructed spectra and avoid misinterpretations associated with the approximation of a single gyro-magnetic ratio used to determine the energy axis of the spectrum. However, it is important to remember that scattering intensities reflect the interference of the molecular wave-function. In particular, for rotating states (*J* > 0) we expect the scattering to strongly depend on the rotational projection state of the incoming state.^31,32,37,38^ To calculate a realistic signal and obtain information about the relative intensity of the inelastic peaks, the actual elements of the scattering matrix are needed. One way, is to try and calculate the elements of the scattering matrix quantum mechanically using multi-dimensional molecule-surface potentials, similarly to what has been done for elastic scattering.^39,40^ While it would be challenging to do this accurately, the signal corresponding to the matrix elements could be compared with the experimental result and would be an excellent benchmark for the accuracy of the theoretical modelling. Another method would be to determine the scattering matrix elements directly from a best fit to the experiment, however, again so far this has only been achieved for elastically scattered molecules.^32^

## Conclusions and outlook

In the current work, we have demonstrated that it is possible to study surface phonons using molecular spin-echo measurements, specifically using D_2_ to study phonon excitations of a Cu(111) surface. At the simplest level, the data can be analysed in the same way as ^3^He spin-echo measurements, using a single gyro-magnetic ratio which corresponds to the frequency that contributes most to the measured signal, which in the case of D_2_ is Δ*m*_*I*_ = 1 transitions in *I* = 2, *J* = 0. The position of the main peaks in the reconstructed spectra are in good agreement with previous TOF measurements^34^ and using optimal tilted projection angles we can measure these excitations with a high energy resolution.

The energy and momentum range, and the energy resolution which can be obtained when measuring phonons with a molecular beam of hydrogen is similar to that obtained with helium spin-echo. Depending on which hydrogen isotope is used the absolute value of the gyro-magnetic ratio can be higher (H_2_) or lower (D_2_) than ^3^He, however, the effects of this on the resolution can be compensated by changing the velocity of the beam and the corresponding time spent in the magnetic field. Unlike quasi-elastic scattering experiments, where fields are ramped up to the limit to obtain sub-μeV energy resolution,^41^ when measuring phonons using spin-echo, the natural width of the phonon peaks is typically wider than tens of μeV. As a result, the signal decays at magnetic field values which are significantly lower than the maximum value, and it is the signal to noise which eventually restricts the resolution. For example, the sampling resolution of the spectrum shown in Fig. 6 is approximately 30 μeV in the vicinity of the energy gain peak. While the current in the solenoid coil could easily be increased by an order of magnitude, dividing the peak into much smaller energy bins would make it too noisy to be useful, unless further averaging is performed. Helium and molecular spin echo also share a common limitation in comparison with time-of-flight HAS. The range of energies for both the incoming and scattered beams, is restricted to energies where the hexapole focussing is efficient, estimated crudely as 5–30 meV for our experimental setup. Hence, when a wide range of energies or large energy gain/loss processes are of interest, time-of-flight HAS is a more suitable choice, whereas both helium and molecular spin echo are superior when it comes to measuring low energy excitation with very high energy resolution.

Our results also demonstrate the complexities of molecular spin-echo, as the reconstructed spectrum appears to contain additional peaks at energies which do not correspond to the expected phonon excitation or de-excitation processes, but originate from other frequencies in the signal, related to the multiple spin and rotational states. As we have shown, this ambiguity can be addressed by calculating spectra from semi-classical simulations and determining whether the peaks in the spectra reconstructed from the measured oscillation curves, are due to inelastic scattering events or due to molecules in other states. The results of the calculation confirm that the largest peaks in our data come from molecules in the *I* = 2, *J* = 0 state scattering inelastically due to energy exchange with the surface phonons, but that the smaller peaks we measured are consistent with scattering of molecules in *I* = 1, *J* = 1. In addition, the simulations reproduce our experimental observation that the peak widths of the different phonons change dramatically for optimal tilted projection angles, confirming that the classical picture of tilted projections, originally developed for helium, is still useful for performing high resolution inelastic scattering experiments of molecules.

It is currently not possible to make a quantitative comparison between the relative intensities in the spectra reconstructed from the experimental data and those reconstructed from semi-classical simulations. The reason for this is that in our simulations we have assumed that the scattering matrix, which characterises how the amplitude of the *m*_*I*_ and *m*_*J*_ states change when the molecules scatter from the surface, is an identity matrix. A realistic calculation will include the probability of exciting a phonon, and also the change of the *m*_*J*_ states for *J* > 0 molecules.

While the existence of rotating states in the beam complicates the analysis, it also represents a particularly interesting direction for future development. When calculations of phonon excitation probabilities and scattering matrices are available, these can be used to simulate spectra, compare with the experimental results, and benchmark the accuracy of the theory used to calculate them. It may also be possible to extract the scattering matrix elements directly from inelastic scattering measurements as we have done previously for elastic scattering of H_2_,^32^ which will provide unique insight into how the interaction of the phonons is influenced by the rotational projection states as these can be obtained simultaneously from a single measurement. It is also important to stress that molecular rotation, and specifically the breaking of symmetry due to change of the *m*_*J*_ states during the molecule-surface collision, should make it possible to detect phonons which cannot be measured in helium scattering measurements, as demonstrated for the case of the shear horizontal phonons of the NaCl surface.^15^

Finally, the simulations that have been used in the current work are semi-classical, in the sense that the centre of mass of the molecules is propagated through the magnetic fields classically and only the nuclear and rotational magnetic moments are propagated quantum mechanically. For the case of elastic scattering, it has been shown that this gives the same results as a fully quantum propagation method up to magnetic field values of 10^4^ gauss,^42^ but the reliability of using this semi-classical approach compared to a full quantum mechanical approach for inelastic scattering is the subject for future research work.

## Funding

This work was funded by an ERC consolidator grant (Horizon 2020 Research and Innovation Programme grant 772228), and an EPSRC New Horizons grant (EP/V048589/1).

## Conflicts of interest

There are no conflicts to declare.

## Supplementary Material
